# The Simillimum

**Published:** 1884-07

**Authors:** J. T. Kent

**Affiliations:** St. Louis


### THE SIMILLIMUM.

PROF. J. T. KENT, A. M., M. D., ST. LOUIS.

I had supposed that this question had been settled, but it seems I am not informed, as many are saying the only thing necessary is to find the name of an agent capable of causing similar symptoms on the healthy and the simillimum is that agent. I cannot accept that as the teaching of the master.

These perverters of truth claim that the self-same agent will cure in any dose or any potency. My statement is that the simillimum, the curative power or force, is not essentially the curative drug. The simillimum may be found in Aconite 200 where Aconite 3x has failed. Then Aconite is the curative agent but not the simillimum, but Aconite 200 is the simillimum. Where Aconite tincture cures, and cures permanently, I believe it does so because it is the simillimum. I have recently seen Arsenicum 200 fail in a case so clearly indicating Arsen. that a tyro could not fail to see it, and the same 200 is known to be genuine and has for years served well; the 8,000 of Jenichen cured promptly. The remedy was Arsenicum, but the simillimum was Arsenicum$^{8m}$. I have seen this same Ars.$^{8m}$ cure when the 3x, 6x, 30, 60, and 200 had failed.

Then the simillimum must be the curative power and not the name of any given drug. I may conclude that Ars. is the remedy and the case is not cured! I must next choose a suitable potency and as suitably refrain from its repetition. The smallest part of the conclusion has been wrought when the name of the curative agent has been decided. I admit it is seldom necessary to be so exclusive in finding the curative power, but that it does sometimes occur I am more than convinced. A friendly doctor said to me a few days ago in my office that he was curing a case of psoriasis with Ars. 3x. He stated that the patient had been taking it off and on for a year, and that when he stopped the medicine the disease seemed to come back. Nothing can be learned about such a case, as there was no clear statement of the facts in the case. But it is so much more satisfactory to use a very high attenuation of any drug believed to represent the curative power in a single dose. It is the safest and surest way to avoid a mistake. If the remedy acts, it is so permanent and almost sure to be the simillimum. If it does not act, there is no harm done and a lower potency may be selected. If a lower potency is selected and repeated, as often has to be, the over-action spoils the case and sometimes precludes the possibility of a cure. If the remedy is homœopathic to a given totality, a single dose very high may cure the whole case; if, however, it seems necessary to repeat, and the disease only disappears while the remedy is being repeated the selection is a bad one and had better be changed.

This knowledge we gain while using a high potency if a given case leads us slowly but surely in the way of success.

It is a grand mistake to fly to a low power because a high has failed to act, yet it may be tried as a manner of convincing man of his own weakness.

The simillimum is the curative power that every true healer is in search of, and I take it for granted that every physician in his heart is searching for truth. Then it must appear to all unprejudiced minds that the name of a drug is no more the curative power than the name of a disease is the disease to be cured. As any given disease has an individuality in causes of varied intensity, so will its cure be in antagonism of varied intensity. One drop of Aconite root may cure the Aconite mental picture in one person and fail signally in many, and the 200 cure the case in a few hours. I would not say *may*, unless I had seen the work.

I had once under my care a patient whose symptoms were like those of Sulphur. As I had not advanced in knowledge beyond the 6x, I gave that remedy in the potency named with what seemed to me astonishing relief. Finally, Sulph. 6x failed to give the continued relief, although the *agent* (for it was not a remedy) was continuously repeated. I compared Sulph. with the patient, and Sulph. seemed still indicated, but it would not cure. I must change!

I changed and changed, and finally the patient changed. I spoiled my case, and felt like "cussing" somebody for it. Nobody to blame but myself. Some three years later this patient, finding nobody that could do any better than I had done, *bad as it was*, came back to me, and by the way I had changed I had opened my eyes; this patient had taken many crude drugs, but I then knew how to develop a case and cure it. He took Nux.$^{2m}$ for a few weeks with improvement, but the same old burning on top of head and soles, the same 11 A. M. hungry stomach, the same itching, and the same "not very well myself" all there. These symptoms had never met the simillimum.

The famous Sulph.$^{55m}$ one single dose and S. L. made astounding changes that lasted for nearly two months, when the returning symptoms were the signal for another dose. Three doses cured the case permanently. Sulph.$^{55m}$ was the *simillimum*, Sulph. 6x was not, therefore Sulph. was not the simillimum. Sulph. was his remedy, but the attenuation was next to be chosen. Why is this not true of any agent in the materia medica? There is nothing new in these facts, but it seems so strange that there can be found a man with brain too small to comprehend it or too dishonest to own it or too skeptical to believe it.

The microcephalic panderers to the loud-mouthed ignoramuses are seeming to rule the world by their mighty majority, but pure Homœopathy has continued to grow and will continue to grow, and the educated, thinking people of the world will support it just as rapidly as they are made acquainted with it. No man shall tie me down to the limits of a microscope or to his own narrow sphere of observation or accepted truth. The man that remains in the lower strata of potential *simillimums* and demands that everybody must worship with him is too narrow to be called a healer or a benefactor of man.

The simillimum may be found in the lowest attenuations, but is positively found for all curable diseases in the high and highest genuine potencies.

